# Provider Perspectives on Pediatric Store-and-Forward Teledermatology at Boston Medical Center: Cross-Sectional Survey

**DOI:** 10.2196/67728

**Published:** 2025-06-02

**Authors:** Mason McDowell, Sara Stulac, Margaret S Lee

**Affiliations:** 1 Boston University Chobanian & Avedisian School of Medicine, Boston, MA, United States; 2 Department of Pediatrics, Boston University Chobanian & Avedisian School of Medicine, Boston, MA, United States; 3 Department of Dermatology, Boston University Chobanian & Avedisian School of Medicine, 609 Albany St, Boston, MA, 02118, United States, 1 6178218308

**Keywords:** teledermatology, residency education, pediatric dermatology access, eConsult, telemedicine, pediatric dermatology

## Abstract

This cross-sectional survey of pediatric dermatology and primary care pediatric providers found that store-and-forward teledermatology is an efficient and educational means of delivering care to a safety-net pediatric patient population.

## Introduction

Access to pediatric dermatologists is limited by prolonged waiting times, limited appointments, and uneven geographic availability [1]. Provider-to-provider store-and-forward teledermatology (SAFTD), which allows referring providers to send images and clinical information to dermatologists for asynchronous evaluation, triage, and recommendations, alleviates these barriers [2,3]. In 2020, Boston Medical Center (BMC) launched an Epic-based SAFTD service for pediatric providers (PPs), with dermatology residents and board-certified pediatric dermatologists responding to requests within 72 hours. We hypothesized that SAFTD is particularly helpful to patients and providers in safety-net hospital (SNH) systems like BMC, where language, transportation, and financial challenges play heightened roles in care delivery [4,5].

## Methods

### Study Design

A cross-sectional REDCap (Research Electronic Data Capture [Vanderbilt University]) survey was emailed to all BMC pediatric SAFTD users, including pediatric dermatologists, dermatology residents, pediatricians, pediatric residents, and pediatric nurse practitioners. Completed responses were collected between June 29 and August 7, 2023. Surveys included qualitative and Likert scale (range 0-5) data, which were examined via thematic and univariate analyses, respectively.

### Ethical Considerations

The survey was anonymous and was approved as exempt by the BMC institutional review board (H-43783). No renumeration was provided.

## Results

Among 15 (58%) responses obtained from 26 PPs, the mean satisfaction score was 4.93 (SD 0.29), with 93% (14/15) reporting they were very satisfied. All PPs preferred SAFTD over traditional referral methods (Table 1), primarily citing decreased time to intervention and saving patients resources (eg, time and cost of travel to clinic). Satisfaction with the response time (mean 4.8, SD 0.4), digital template (mean 4.33, SD 1.07), and time to face-to-face visit (mean 4.26, SD 0.93) was high. Recommendations were communicated via phone for 66% (10/15) of PPs, with 33% (5/15) using the patient portal. Barriers to using SAFTD included difficulty capturing high-quality photographs, providers’ limited time for contacting patients regarding recommendations, and challenges with uploading photographs. PPs appreciated the opportunity to learn dermatology in real time, and 93% (14/15) reported changing their subsequent patient management practices after using SAFTD.

**Table 1.** Pediatric provider responses to multiple-choice questions. The total number of pediatric provider respondents was 15.

Questions and respondents’ selections	Respondents, n (%)
How satisfied are you with the Pediatric Dermatology eConsult^a^ service?^b^	
4	1 (7)
5	14 (93)
How satisfied are you with the response time for pediatric dermatology eConsults?^b^	
4	3 (20)
5	12 (80)
How satisfied are you with the template used to provide content in the pediatric dermatology eConsults?^b^	
1	1 (7)
3	1 (7)
4	4 (27)
5	9 (60)
How satisfied are you with the referral time to in-person evaluation, if indicated, by pediatric dermatology eConsult?^b^	
2	1 (7)
3	2 (13)
4	4 (27)
5	8 (53)
What proportion of eConsult diagnoses are concordant with your initial evaluation?^c^	
1	1 (7)
2	1 (7)
3	4 (27)
4	7 (47)
5	2 (13)
Has the information provided in the pediatric dermatology eConsult(s) changed the way you manage other patients?	
No	1 (7)
Yes	14 (93)
What impact has the information provided in the pediatric dermatology eConsults had on the number of patients you refer to dermatology?	
Decreased	4 (27)
No change	3 (20)
Increased	8 (53)
Do you prefer the eConsult or traditional referral system?	
eConsult	15 (100)
With what method do you inform patients of recommendations made via eConsult?	
Patient portal	5 (33)
Phone call	10 (67)

a “eConsult” is synonymous with provider-to-provider store-and-forward teledermatology.

b Likert scale: 1=not satisfied at all; 5=the most satisfied.

c Likert scale: 1=always discordant; 5=always concordant.

Among 7 (41%) responses obtained from 17 dermatology providers (DPs), the mean satisfaction score was 4 (SD 0.53), and 86% (6/7) preferred SAFTD over traditional referral methods (Table 2), citing improved triage and decreased time to intervention. Additional SAFTD benefits included increased collaboration between dermatology and pediatrics departments and decreased language barriers. DP priorities for photograph improvement included focus, lighting, and an adequate number of photos and views. Patients’ and guardians’ comprehension of teledermatology recommendations at follow-up in-person appointments was assessed by DPs as moderate overall (mean 2.67, SD 0.51). One respondent preferred traditional referrals, stating that in-person assessment is necessary for adequate diagnoses and that SAFTD may delay appropriate care.

**Table 2.** Dermatology provider responses to multiple-choice questions. The total number of dermatology provider respondents was 7.

Questions and respondents’ selections	Respondents, n (%)
How satisfied are you with the Pediatric Dermatology eConsult^a^ service?^b^	
3	1 (14)
4	5 (71)
5	1 (14)
To what extent do eConsults include adequate quality photos?^c^	
2	1 (14)
3	3 (43)
4	3 (43)
Rate the importance of the below actions for eConsult quality	
Checking that images are in focus^d^	
5	7 (100)
Making sure images have clear orientation and location^d^	
3	3 (43)
4	1 (14)
5	3 (43)
Including images of symmetrical contralateral skin for rashes^d^	
2	2 (29)
3	3 (43)
4	1 (14)
5	1 (14)
Ensuring adequate lighting^d^	
4	3 (43)
5	4 (57)
Verifying adequate number of photos/views^d^	
3	1 (14)
4	4 (57)
5	2 (29)
Making sure hair does not obscure condition^d^	
3	2 (29)
4	4 (57)
5	1 (14)
Do eConsults include adequate clinical information?^c^	
2	1 (14)
3	4 (57)
4	2 (29)
What is your overall level of comfort diagnosing conditions via eConsult?^e^	
3	3 (43)
4	4 (57)
To what extent do eConsults reduce unnecessary face-to-face dermatology appointments?^f^	
3	5 (71)
4	2 (29)

a “eConsult” is synonymous with provider-to-provider store-and-forward teledermatology.

b Likert scale: 1=not satisfied at all; 5=the most satisfied.

c Likert scale: 1=none of the time; 5=all the time.

d Likert scale: 1=not important; 5=very important.

e Likert scale: 1=unable to make diagnoses via eConsult; 5=equally comfortable diagnosing as compared to face-to-face visits.

f Likert scale: 1=not at all; 5=greatly.

## Discussion

BMC PPs and DPs are highly satisfied with SAFTD, particularly with its ability to facilitate prompt treatment, superior triage, and reduced barriers to care. Providers highlighted that it decreases language and transportation barriers, which disproportionately affect the diverse and low-income populations served by BMC [4,5]. The proportion of BMC PPs “very satisfied” with SAFTD surpasses those reported in similar studies conducted in non-SNH settings [6,7]. Although participation bias may have contributed, PPs’ high satisfaction with SAFTD suggests heightened value in SNHs. These findings support literature showing that SAFTD increases access to care [2,3,8].

Previous studies noted photograph quality as an SAFTD limitation [3,6]. Our results provide more details for improving pediatric photography support or training to ensure images are in focus, are taken with adequate illumination, and provide a comprehensive range of clinical views to improve SAFTD quality.

Our study shows that SAFTD can be used to train pediatric and dermatology residents, as well as PPs with less dermatology experience. PPs valued how SAFTD facilitated real-time learning in dermatology; 93% affirmed that the service improved their management practices for dermatological conditions. This shows how learning co-occurs with routine patient care, supporting quiz-based data and survey studies suggesting that PPs learn from SAFTD over time [8,9]. In the context of variable dermatological training for pediatricians [10] and pediatric advanced practice providers, SAFTD presents an opportunity to incorporate dermatology education into day-to-day primary care. With respect to mitigating board-certified pediatric dermatologist workforce shortages [1], increasing PPs’ knowledge of common dermatological conditions allows DPs to focus on complex and severe conditions.

We provide evidence that SAFTD may be particularly helpful for pediatric residency programs and SNHs serving resource-limited populations. Addressing PPs’ barriers to SAFTD use by providing photography support and allocating protected time for dermatologic recommendations would strengthen SAFTD’s benefits. Efforts to optimize SAFTD for primary care, dermatology education, and expanded pediatric dermatology access hold significant promise.

## Supplementary material

10.2196/67728 Multimedia Appendix 1 Supplementary data and information files.

## Abbreviations:

BMC: Boston Medical Center
DP: dermatology provider
PP: pediatric provider
REDCap: Research Electronic Data Capture
SAFTD: store-and-forward teledermatology
SNH: safety-net hospital
